# Supplementary material for: Identification of the cellular factor KLF16 as a novel epigenetic repressor of HIV-1 transcription
Source: Res Sq. 2026 Jun 4:rs.3.rs-9683098. Preprint. [Version 1] doi: 10.21203/rs.3.rs-9683098/v1 (PMC13252555; doi:10.21203/rs.3.rs-9683098/v1)
Supplement: 1 [file NIHPPRS9683098V1-supplement-1.pdf]

SUPPLEMENTARY TABLE 1.

REAGENT or RESOURCE	SOURCE	IDENTIFIER
**Cell lines**		
Jurkat	AIDS Research and Reference Reagent Program	RRID: CVCL_0065
THP1	AIDS Research and Reference Reagent Program	CVCV_0006
Jurkat HIVGKO	This paper	N/A
THP HIVGKO	This paper	N/A
Jurkat pLVX-KLF16	This paper	N/A
Jurkat pLVX-empty	This paper	N/A
HEK293T	American Type Culture Collection (ATCC, Manassas, VA)	RRID: CVCL_0063
**Antibodies**		
Purified NA/LE Mouse Anti-Human CD3 (Clone UCHT1)	BD	Cat#555329
Purified NA/LE Mouse Anti-Human CD28 (Clone CD28.2)	BD	Cat#555725
HIV-1 core (p24) antigen-RD1 (Clone KC57)	Beckman Coulter	Cat# 6604667
Mouse anti-human CD8 FITC	Miltenyi	Cat#130-113-157
Mouse anti-human CD3 Pacific Blue (Clone UCHT1)	BD	Cat#558117
Mouse anti-human CD4 AF700 (Clone RPA-T4)	BD	Cat#557922
Anti-human CD1c (BDCA-1) Phycoerythrin (Clone REA694)	Miltenyi	Cat#130-110-536
Anti-human HLA-DR Brilliant Violet 785 (Clone L243)	Biolegend	Cat#307642;
Mouse anti-human CD14 APC (Clone M5E2)	BD	Cat#555399
Mouse anti-human CD16 Phycoerythrin-Cyanine 7 (Clone 3G8)	BD	Cat#560918
LIVE/DEAD Fixable Aqua Dead Cell Stain Kit (405 nm excitation)	Invitrogen	Cat#L34957
IgG Rabbit	Cell Signalling Technology	Cat#2729S
Anti-H3	Cell Signalling Technology	Cat#4620
Anti-H3K9ac	Cell Signalling Technology	Cat#9649
Anti-H3K27ac	Cell Signalling Technology	Cat#8173
Anti-H3K9me3	Cell Signalling Technology	Cat#13969

Anti-RNA Pol II	Diagenode	Cat#C15200004
Anti-Sp1	Millpore	Cat#07-645
Anti-HDAC1	Cell Signalling Technology	Cat#34589
**Bacterial and Virus Strains**		
VSV-G Plasmid	National Institution of Health (NIH)	Cat#ARP-4693
NL4.3BaLDenv GFP	National Institution of Health (NIH)	Cat#ARP-12637
HIV 89.6	AIDS Research and Reference Reagent Program	Cat#ARP-12485
HIVGKO	Provided by Eric Verdin (Buck Institute for Research on Aging, USA)	N/A
pNL4.3GFP	AIDS Research and Reference Reagent Program	Cat#ARP_11349
pcDNA3.1	Thermo Fisher	Cat#V79020
pHa-KLF16	This paper	N/A
pSp1	Addgene	Cat#232649
pLTR-Fluc	Published by our goup previously (Verdikt et al., 2022)	N/A
pLTR-FlucΔSp1	This paper	N/A
pLTR-FlucΔSp1Leader	This paper	N/A
pLVX-empty	This paper	N/A
pLVX-KLF16	This paper	N/A
shCTL	Vector builder	Cat#VB900126-3570CSV
shKLF16	Vector builder	Cat#VB10000-0013dtn
shSp1	Horizon discovery	Cat#RHS3979-201751561
pVSG-G	Provided by Angela Ciuffi (University of Lausanne, Switzerland)	N/A
psPAX2	Provided by Angela Ciuffi (University of Lausanne, Switzerland)	N/A
**Biological Samples**		
Leukaphereses of ART treated and untreated people living with HIV	Recruited at the Montreal Chest Institute, McGill University Health Centre and Centre Hospitalier de l'Université de Montréal with the help of Dr Jean-Pierre Routy's group	N/A
**Chemicals, Peptides, and Recombinant Proteins**		
rhIL-2	R&D Systems	Cat#202-IL-050
T Cell TransAct	Miltenyi Biotec	Cat#130-111-160

M-CSF	Proteintech	Cat#HZ-119
GM-CSF	Proteintech	Cat#HZ-1002
Recombinant Human Macrophage Colony Stimulating Factor (M-CSF)	R&D Systems	Cat#216-MC-025
DMEM, high glucose, GlutaMAX™ Supplement	Thermo Fisher	Cat#10566016
RPMI 1640 Medium (RPMI)	Thermo Fisher	Cat# 11875119
Lymphocyte Separation Medium (LSM)	Wisent	Cat#305-010-CL
Penicillin/Streptomycin	Gibco	Cat# 15140-122
Fetal Bovine Serum (FBS)	Wisent	Cat#091-150
Bovine Serum Albumin (BSA)	BioShop	Cat# ALB001
Phosphate Buffered Saline (PBS)	Thermo Fisher	Cat# 20012050
Tween 20	Thermo Fisher	Cat# BP337-500
Triton X-100	Sigma	Cat#X100-500mL
Dimethyl Sulfoxide (DMSO)	Sigma	Cat#34869-500mL
UltraComp eBeads Plus	Thermo Fisher	Cat# 01-3333-42
Trypan Blue	Thermo Fisher	Cat#15250061
Bovine Serum Albumin (BSA)	BioShop	Cat#ALB001.500
Streptavidin Horseradish Peroxidase (Strep-HRP)	Fisher Scientific	Cat#65R-S104PHRP
3,3',5,5'-Tetramethylbenzidine (TMB)	Quimigen	Cat#42R-TB10265R-S104PHRP
Phosphoric Acid (H_3PO_4)	Sigma	Cat#PX0996
Formaldehyde solution 37 wt. % in H2O	Sigma	Cat#F1635-500ML
Sodium Azide	Bioshop	Cat#SAZ001.250
2-Mercaptoethanol	Sigma	Cat#M6250
Tris HCl	BioShop	Cat#TRS002.500
Proteinase K	Fisher Scientific	Cat#25530-015
Molecular Grade Water (H_2O)	Wisent	Cat#809-115-CL
10X PCR Buffer / Magnesium Chloride ($MgCl_2$) Buffer / Thermus Aquaticus (TAQ) Polymerase	Thermo Fisher	Cat#18038067
LC480 probe master mix	Roche	Cat#4707494001
Deoxynucleoside Triphosphates (dNTP)	Thermo Fisher	Cat#10297018
All-trans Retinoic Acid (ATRA)	Sigma	Cat#R2625-50MG
cOmplete™, Mini, EDTA-free Protease Inhibitor Cocktail	Roche	Cat#11836170001

Puromycin	Sigma-Aldrich	Cat#P8833
**Critical Commercial Assays**		
Memory CD4+ T Cell Isolation Kit, human	Stemcell	Cat# 19157
Pan Monocyte Isolation Kit	Miltenyi	Cat#130-096-537
Fixation/Permeabilization Solution Kit (Cytofix/Cytoperm)	BD	Cat#554714
p24 ELISA	Homemade. Hybridome provided by Dr. Michel J. Tremblay (Bounou et al., 2002)	N/A
Human T Cell Nucleofector® Kit	Lonza	Cat# VPA-1002
All Prep DNA/RNA/miRNA Universal Kit	Qiagen	Cat#80224
QuantiTect SYBR Green RT-PCR Kit	Qiagen	Cat #204245
Rneasy Plus Mini Kit	Qiagen	Cat#74136
NEBuilder HiFi DNA Assembly Mix	New england biolabs	Cat #E2621L
TurboFect transfection reagent	Thermo Fisher	Cat #R0533
Calphos transfection reagent	Takara	Cat#631312
SingleGlo luciferase reporter assay	Promega	Cat#E1500
Bio-Rad Protein Assay Kit I	Bio-Rad	Cat#5000001
All-In-One DNA/RNA/Protein Miniprep Kit	Biobasic	Cat#BS88003
HiScript III RT SuperMix for qPCR	Vazyme	Cat#R323-01
Luna Universal qPCR Master Mix	New england biolabs	Cat#M3003S
DC™ Protein Assay Kit II	Bio-Rad	Cat##5000112
Lymphoprep	Stemcell	Cat#18061
EasySep™ Human CD4+ T Cell Isolation Kit	Stemcell	Cat#17952
EasySep™ Human Monocyte Isolation Kit	Stemcell	Cat#19359
T Cell TransAct	Miltenyi Biotec	Cat#130-111-160
**Oligonucleotides**		
SmartPool ON-TARGETplus Human KLF16 5nmol	Horizon Dharmacon	L-007083-00-0005
ON-TARGETplus Non-targeting Control Pool 5nmol	Horizon Dharmacon	D-001810-10-05
**Software and Algorithms**		
Graphpad prism 10	Graphpad	www.graphpad.com

R studio	The R Project for Statistical Computing	https://www.r-project.org/
FlowJo version 10	BD	https://www.flowjo.com/
**Others**		
MACS LS Columns	Miltenyi	Cat# 130-042-401
Pre-Separation Filters 30 µm	Miltenyi	Cat# 130-041-407

Supplementary Table S1: List of reagent and resources used in this study

SUPPLEMENTARY TABLE 2.

**DNA pull down**	
**DNA pulldown probe (Sens)**	5'-/5deSBioTEG/TTTCCGCTGGGGACTTTCC AGGGAGGTGTGGCCTGGGCGGGACTG GGGAGTGGCGAGCCCTCAGATGCTAC ATATAAGCAGCTGCTTTTTGCCTGTACTG - 3'
**DNA pulldown probe (AntiSens)**	5'-/5Cy3/CAGTACAGGCAAAAAGCAGCTGCTTAT ATGTAGCATCTGAGGGCTCGCCACTCCCCAGTCCCGCC CAGGCCACACCTCCCTGGAAAGTCCCCAGCGGAAA- 3'
**EMSA probe**	
**Sp1 III (sens)**	5'CGGGACTGGGGAGTGGCGAGCCCTC3'
**Sp1 III (antisens)**	5'GAGGGCTCGCCACTCCCCAGTCCCG3'
**Sp1 II (sens)**	5'TTTCCAGGGAGGCGTGGCCTGGG3'
**Sp1 II (antisens)**	5'CCCAGGCCACGCCTCCCTGGAAA3'
**SP1 I (sens)**	5'TGGCCTGGGCGGGACTGGGGAGT3'
**Sp1 I (antisens)**	5'ACTCCCCAGTCCCGCCCAGGCCA3'
**5'LTR Sp1 (I-III) (sens)**	5'-CCAGGGAGGCGTGGCCTGGG CGGGATGGGGAGTGGCGAGC-3'
**5'LTR Sp1 (I-III) (antisens)**	5'-GCTCGCCACTCCCCAGTCCCG CCCAGGCCACGCCTCCCTGG-3'
**Cloning primers**	
**pHa-KLF16 (sens)**	5'-GCAGATATCCAGCACAGTGGCTGAT ATCGAATTCCTGCAGCCCG-3'
**pHa-KLF16 (antisens)**	5'-GGGTTTAAACGGGCCCTCTAGA GAAGTTTGTTGCGCGGATC-3'
**pLTR-FlucΔSp1 and pLTRFlucΔSp1Leader (sens)**	5'-GATCGATCCTCTAGCCCGGGCGGGAGGTACGTGGAAGG GCTAATTCACTCCC-3'
**pLTR-FlucΔSp1 and pLTRFlucDSp1Leader (antisense)**	5'GCCAAGCTTACTTAGATCGCATCGAGCTAGACTCTCTCCTTCT AGCCTCC-3'
**pLVX-KLF16 (sens)**	5'-TAGAGGATCTATTTCCGGTGGCCACCATGGACTACAAAGA-3'
**pLVX-KLF16 (antisens)**	5'-GGGAGGGAGAGGGGCGGTCAGCCGGATCCAAGCCCGGCAG-3'

**PCR and qPCR primer for quantification of HIV integrated DNA**	
CD3 external primer 1	5’-ACTGACATGGAACAGGGGAAG-3’
CD3 external primer 2	5’- CCAGCTCTGAAGTAGGGAACATAT-3’
Primer GagR	5’- AGCTCCCTGCTTGCCCATA-3’
Primer Alu1	5’- TCCCAGCTACTGGGGAGGCTGAGG-3’
Primer Alu2	5’- GCCTCCCAAAGTGCTGGGATTACAG-3’
Primer LM667	5’- ATGCCACGTAAGCGAAACTCTGGCTAACTAGGGAACCCA CTG-3’
Primer LambdaT	5’- ATGCCACGTAAGCGAAACT-3’
Primer AA55M	5’- GCTAGAGATTTTCCACACTGACTAA-3’
CD3 internal primer 1	5’- CCTCTCTTCAGCCATTTAAGTA-3’
CD3 internal primer 2	5’- GGCTATCATTCTTCTTCA AGG T-3’
Probe LTR-LC	5’-LC640-CACTCAAGGCAAGCTTTATTGAGGC-3’-phosphate
Probe LTR-FL	5’- CACAACAGACGGGCACACACTACTTGA-3’-Flurescein
Probe P1	5’-GGCTGAAGGTTAGGGATACCAATATTCCTGTCTC-3’-Flurescein
Probe P2	5’-LC640- CTAGTGATGGGCTCTTCCCTTGAGCCCTTC-3’-phosphate

Supplementary Table S2: List of oligonucleotides used in this study

SUPPLEMENTARY TABLE 3

Participant ID	Sex	CD4 counts$^{\#}$	CD8 counts$^{\#}$	Plasma viral load$^{\&}$	Time since infection*	ART
**Donor #1**	M	398	775	<40	154	Complera
**Donor #2**	M	542	803	<40	13	Stribild
**Donor #3**	M	743	899	<40	171	Truvada/Raltegravir
**Donor #4**	M	841	1322	<40	149	Sustiva/Truvada
**Donor #5**	M	908	854	<40	89	Stribild
**Donor #6**	M	963	644	<40	123	Prevista/Kivexa/Norvir
**Donor #7**	M	288	407	<40	211	Atripla
**Donor #8**	M	649	620	<40	186	Triumeq

$^{\#}$, cells/µl; $^{\&}$, HIV RNA copies per ml plasma; *, months; ART, antiretroviral therapy; $^{\$}$, months; NA, information not available; ND, not detected

Supplementary Table S3: Clinical parameters of HIV-infected study participants receiving viral suppressive antiretroviral therapy (ART).
